# Resprouters Versus Reseeders: Are Wild Rooibos Ecotypes Genetically Distinct?

**DOI:** 10.3389/fgene.2021.761988

**Published:** 2021-12-20

**Authors:** J. Brooks, N. P. Makunga, K. L. Hull, M. Brink-Hull, R. Malgas, R. Roodt-Wilding

**Affiliations:** ^1^ Department of Botany and Zoology, Stellenbosch University, Matieland, South Africa; ^2^ Department of Genetics, Stellenbosch University, Matieland, South Africa; ^3^ Department of Conservation Ecology and Entomology, Stellenbosch University, Matieland, South Africa

**Keywords:** genetic diversity, medicinal plants, microsatellites, phylogeography, population genetic structure, rooibos, wild populations

## Abstract

*Aspalathus linearis* (Burm. F.) R. Dahlgren (Fabaceae) or rooibos, is a strict endemic species, limited to areas of the Cederberg (Western Cape) and the southern Bokkeveld plateau (Northern Cape) in the greater Cape Floristic Region (CFR) of South Africa. Wild rooibos, unlike the cultivated type, is variable in morphology, biochemistry, ecology and genetics, and these ecotypes are broadly distinguished into two main groups, namely, reseeders and resprouters, based on their fire-survival strategy. No previous assessment of genetic diversity or population structure using microsatellite markers has been conducted in *A. linearis*. This study aimed to test the hypothesis that wild rooibos ecotypes are distinct in genetic variability and that the ecotypes found in the Northern Cape are differentiated from those in the Cederberg that may be linked to a fire-survival strategy as well as distinct morphological and phytochemical differences. A phylogeographical and population genetic analyses of both chloroplast (*trn*LF intergenic region) and newly developed species-specific nuclear markers (microsatellites) was performed on six geographically representative wild rooibos populations. From the diversity indices, it was evident that the wild rooibos populations have low-to-moderate genetic diversity (He: 0.618–0.723; Ho: 0.528–0.704). The Jamaka population (Cederberg, Western Cape) had the lowest haplotype diversity (*H* = 0.286), and the lowest nucleotide diversity (*π* = 0.006) even though the data revealed large variations in haplotype diversity (*h* = 0.286–0.900) and nucleotide diversity (*π* = 0.006–0.025) between populations and amongst regions where wild rooibos populations are found. Our data suggests that populations of rooibos become less diverse from the Melkkraal population (Suid Bokkeveld, Northern Cape) down towards the Cederberg (Western Cape) populations, possibly indicative of clinal variation. The largest genetic differentiation was between Heuningvlei (Cederberg, Western Cape) and Jamaka (F_ST_ = 0.101) localities within the Cederberg mountainous region, and, Blomfontein (Northern Cape) and Jamaka (Cederberg) (F_ST_ = 0.101). There was also a significant isolation by distance (R^2^ = 0.296, *p* = 0.044). The presence of three main clusters is also clearly reflected in the discriminant analysis of principal components (DAPC) based on the microsatellite marker analyses. The correct and appropriate management of wild genetic resources of the species is urgently needed, considering that the wild Cederberg populations are genetically distinct from the wild Northern Cape plants and are delineated in accordance with ecological functional traits of reseeding or resprouting, respectively. The haplotype divergence of the ecotypes has also provided insights into the genetic history of these populations and highlighted the need for the establishment of appropriate conservation strategies for the protection of wild ecotypes.

## Introduction

*Aspalathus linearis* (Burm. F.) R. Dahlgren (Fabaceae), is a commercially important South African legume, and a strict endemic of the Cape Floristic Region (CFR). It is more popularly known for its production of rooibos tea, an herbal beverage traditionally harvested in the wild, and now commercially produced for the global export market (Hawkins et al., 2011; Joubert and de Beer, 2011; Van Wyk and Gorelik, 2017). It occurs naturally in the Cederberg region of the Western Cape and in a few areas of the south-western parts of the Northern Cape (e.g., the Suid Bokkeveld and the Noord Bokkeveld Plateau near the rural town of Nieuwoudtville). There are populations within the distribution range that consist of different wild ecological types (ecotypes) (Van der Bank et al., 1999). Variable colour morphs are displayed by wild rooibos populations as the needle-like leaves between populations may range from a light grey-green to a bright green. These ecotypes vary in size/height of the plant, branching structure, leaf size, leaf colour, and leaf and stem thickness (Malgas et al., 2011). *Aspalathus linearis* is particularly important for its role in nitrogen-fixing in N- and P-limited fynbos environments. The fynbos region is a unique biome featuring over 7,000 species that are found in the Western and some parts of the Eastern provinces of South Africa. There are three main plant families that show a high level of species radiation and richness within the fynbos region namely, Restionaceae, Proteaceae and Ericaceae and nutrient poor soils of that are found in the fynbos biome are thought to have led to the high diversity of the Fabaceae plants in this region (Rebelo et al., 2006). It is also a pioneer species in a fire-prone vegetation type, relying on either resprouting from the underground lignotuber of burnt parent plants (resprouters), or as fire-triggered germination of new individuals (reseeders). Congeneric fire-survival strategies are common in several Fynbos taxa, e.g., Proteaceae; Ericaceae and Fabaceae (Marais et al., 2014; Pausas and Keeley, 2014). Rooibos exhibits a plethora of health benefits and is widely used for commercial products such as tea, food products and cosmetics as it has powerful antioxidant properties due to the abundance of flavonoids and other phenolic compounds found throughout the plant (Van Heerden et al., 2003; Smith and Swart, 2018; Bond and Derbyshire, 2020). The commercial importance of rooibos and the value rooibos provides to the livelihood to the local farmers, thus provides further impetus in understanding phylogeographic patterns that are linked to both its metabolites and population genetic structure and evolutionary history (Feliner, 2014). Combined phylogeographic and population genetic level research may also provide useful information for conservation studies by highlighting spatial conservation priorities, and broadening the scope of genetic diversity amongst wild ecotypes, protecting species diversity, similar to studies of rare and endangered species (Pollock et al., 2015; Médail and Baumel, 2018).

Over the past decade, some population genetic studies have been conducted within the Fabaceae family of plants, focusing on *Astralagus bibullatus* (Barneby and E. L. Bridges), *Anthonotha macrophylla* (P. Bauv), and commercial *Cyclopia* species (Baskauf and Burke, 2009; Demenou and Hardy, 2017; Potts, 2017; Niemandt et al., 2018; Galuszynski and Potts, 2020). Van der Bank et al. (1995) studied the genetic variation of wild *A. linearis* and the relationship of four geographically isolated populations, to determine levels of genetic variation and genetic differentiation using isozyme analysis. The study of Van der Bank et al. (1999), also based on isozyme analyses, concluded that resprouters likely evolved from reseeder plants and that this life history strategy was set at the population level. The ecological research of Hawkins et al. (2011) which included more extensive population surveys in the Cederberg showed no overlap between reseeding and resprouting populations in this particular region. However, the influence of the two fire survival strategies of reseeding or resprouting on the genetic diversity of rooibos still remains largely unknown.

Apart from its value in the commercialisation of rooibos, research has also contributed significantly to the understanding of rooibos ecotype diversity, genetic variation and the evolutionary history of wild rooibos (Joubert and de Beer, 2011). Edwards et al*.* (2008) investigated the barcoding potential of three DNA regions for the genus *Aspalathus*. These included nuclear ribosomal *ITS*, plastid *psb*A—*trn*H and *trn*T—*trn*L intergenic regions. Overall, the *trn*Tv*trn*L region was the most discriminatory between the *Aspalathus* species. Very few studies have investigated the complexity and variability between wild rooibos populations on a molecular level and this may be due to morphology, fire-survival strategy, reproductive strategy and biochemical variability (Dahlgren, 1968). The comparative study by Malgas et al. (2010) assessed haplotype variation and morphological variation among wild rooibos populations, using chloroplast *trn*L^UAA^F—*trn*F^GAA^, *trn*T^GGU^—*trn*D^GUC^F, *trn*S^GCU^—*trn*G^UCC^, *trn*T^UGU^F—*trn*L^UAA^R intergenic regions and a nuclear marker, *PIII-PIV*, and observed a correlation between morphology and haplotypic variation. It was speculated that a genetic basis for the observed differences in morphology was important in the inherent morphotypes that are known to occur in the wild, and that are popularly reflected in the local ecological knowledge amongst resource-users (Malgas et al., 2011). The authors postulated that genetic differences between resprouter and reseeder types may play a significant role in the diversification of *A. linearis* as a whole.

There is currently no study that has taken a complementary phylogeographic and population genetics approach in evaluating genetic variability between wild rooibos populations. This study was successful in investigating the phylogeography and population genetics of six wild rooibos ecotypes using both chloroplast sequencing and microsatellite marker analyses. This combined study approach provides foundational genetics research that is novel and may be added to the body of knowledge on rooibos. There are few genetics studied previously performed on rooibos yet wild rooibos ecotypes are well characterised in terms of metabolomic profiles. These ecotypes demonstrate metabolite variability that are linked to geographic localities (Lötter and le Maitre, 2014; Stander et al., 2017; Brooks, 2021). Additionally, the combination of genetic analyses with metabolomics may provide novel insights into understanding the rooibos species. This diversity of ecotypes may be an opportunity for novel products within the rooibos industry; moreover, ecotype diversity may be considered an advantage in the face of climate change (Lötter and le Maître, 2014). By maintaining competitive diversity between ecotypes, wild species are at lower risk of needing protection as microclimates continue to change and populations decline. It is important to emphasise that genetic diversity, even in strict endemic species such as *A. linearis,* is important for long-term conservation planning and for ensuring future sustainability of wild populations. This is because inherent genetic diversity facilitates better adaptation to changing environments, allowing for better population fitness (Potts, 2017). Populations that are continuously declining, often result in reduction in genetic variation, and may lead to inbreeding and/or genetic drift which ultimately reduces the natural fitness and potential adaptability of plants (Baskauf and Burke, 2009).

**FIGURE 1:**
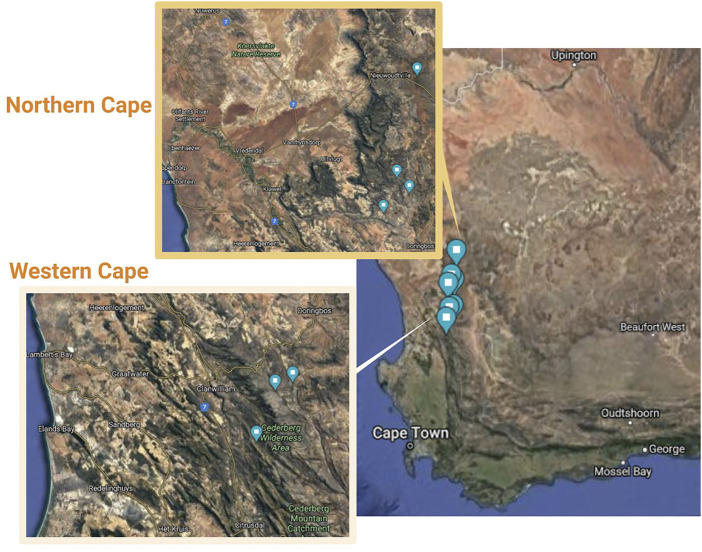
Collection sites of wild-growing *Aspalathus linearis* in the Cederberg area of the Western Cape and Northern Cape of South Africa.

For these reasons, phylogeographic and population level analyses were conducted in this study using both chloroplast DNA (*trn*LF) sequencing analysis and a panel of 11 nuclear microsatellite marker loci to investigate wild populations of *Aspalathus linearis.* This dual-marker approach was chosen as it would allow for a historical and a contemporary assessment of species diversity and genetic differentiation (Wang et al., 2019). This study aimed to test the hypothesis that wild rooibos ecotypes are variable and distinct in genetic variability at the intra- and inter-population levels, and to discriminate wild ecotypes from various geographical regions. This was achieved through the collection of wild accessions from Cederberg in the Western Cape and Nieuwoudtville in the Northern Cape (Figure 1) before investigating the genetic diversity within and between the collected ecotypes using a dual-marker approach.

## Methods and Materials

### Plant Material

Collections of rooibos wild plants were gathered from four localities in Nieuwoudtville and in the Suid Bokkeveld in the Northern Cape with permission from the Heiveld Co-operative and land owners (Table 1; Figure 1). Field harvests were conducted in mid-February 2018. Field guides from local communities assisted with the identification of ecotypes of these plants and these were verified by a botanist (Nokwanda Pearl Makunga). Accessions from the Western Cape were also collected in the Cederberg mountainous region with a flora collection permit issued by CapeNature (Permit number: CN35-28-268) at two locations (Table 1). Branches with leaves near the top of the plant were collected and used for genetic analysis. The individuals that were collected per population ranged from 11 to 15 and were never mixed with other individuals. All samples were placed in individually labelled plastic Ziploc® bags with silica gel granules. These samples were stored in the dark at room temperature until further analysis. These populations can also be distinguished by their fire survival strategies, namely resprouters and reseeders. The Cederberg (Western Cape) populations are typically of the reseeder type, while the Northern Cape populations are commonly of the resprouter type (Malgas et al., 2010). In total, the collected populations cover a distance of 100 km. Representative voucher specimens were deposited in the herbarium of the Department of Botany and Zoology, at Stellenbosch University after confirmation of their taxonomic identity (Table 1).

**TABLE 1:** Wild rooibos (*Aspalathus linearis*) sampling sites and details of collections from the Cederberg region of the Western Cape and the Suid Bokkeveld of the Northern Cape.

Sample site/region	GPS coordinates	Number of individuals	Elevation (m)	Voucher	Fire-survival strategy (resprouter/reseeder)	Distance to nearest town
Heuningvlei, Cederberg	32°12′ S 19°05′ E	11	868	*A.lin*_H2018	Reseeder	67.3 km to Clanwilliam, Cederberg
Jamaka, Cederberg	32°21′ S 19°02′ E	15	405	*A.lin*_J2018	Reseeder	23.8 km to Clanwilliam, Cederberg
Blomfontein, Nieuwoudtville	31°73′ S 19°13′ E	15	740	*A.lin*_B2018	Resprouter	47.4 km to Nieuwoudtville, Northern Cape
Dobbelaarskop, Nieuwoudtville	31°47′ S 19°11′ E	15	718	*A.lin*_D2018	Resprouter	54 km to Nieuwoudtville, Northern Cape
Matarakopje, Nieuwoudtville	31°94′ S 19°11′ E	15	480	*A.lin*_Ma2018	Resprouter	63.5 km to Nieuwoudtville, Northern Cape
Melkkraal, Nieuwoudtville	31°37′ S 19°21′ E	15	780	*A.lin*_M2018	Resprouter	11.5 km to Nieuwoudtville, Northern Cape

### DNA Extraction

Total genomic DNA was extracted from collected leaf material preserved in silica gel, according to a CTAB protocol described by Borse et al. (2011) with specific modifications in order to optimise genomic DNA (gDNA) quality from wild rooibos. This was important as the phenolics of rooibos could potentially influence downstream applications; the modifications are described below. The extraction buffer [10 ml; 2% (w/v) Cetyl Trimethyl Ammonium Bromide (CTAB); 100 mM Tris; 20 mM Ethylenediaminetetraacetic Acid (EDTA); 1.4 M NaCl, with added 2% (w/v) Polyvinylpyrrolidone (PVP)] was preheated in a water bath at 65°C (Sigma Aldrich®). Plant tissue (100 mg) was ground into a fine powder in liquid nitrogen using a mortar and pestle and 1 ml pre-warmed extraction buffer was immediately added to the ground plant material (2 ml Eppendorf tube per reaction). After 20 min, 2 µL of β-mercaptoethanol was added and further incubated at 65°C for 1 h and 30 min. The mixture was then thoroughly vortexed and placed at 65°C for another 20 min heating period before the tubes were cooled down to room temperature and centrifuged at 11,000 rpm (Microlitre centrifuge, Mikro 120, Hettich Zentrifugen) for 10 min. The supernatant was transferred into a new tube, whereas the pellet with the cellular debris was discarded. Chloroform isoamyl alcohol (24:1; v/v) was added to the supernatant. The tubes were again centrifuged at 11,000 rpm for 10 min at 4°C and thereafter, the upper aqueous phase was transferred to new tubes and twice the volume of molecular grade absolute ethanol (BioUltra, Sigma -Aldrich®) was added to precipitate the DNA at −20°C overnight. To collect the DNA, all samples were centrifuged at 11,000 rpm for 10 min at 4°C before the supernatant was discarded and left to dry at room temperature. Once dried, the pellet was dissolved in 60 µL of Tris-EDTA (TE) buffer (10 mM, 1 mM, pH 8.0). The extracted DNA was quantified using a Nanodrop spectrophotometer (NanoDrop™ Lite, Thermo Fischer Scientific). Samples were diluted to a concentration of 50 ng/µL using sterile dH_2_0 and stored at −20°C until further use. The DNA was visualised on a 1% (m/v) agarose gel (6 µL EtBr) to confirm the presence of high-quality DNA.

### Sequencing of Chloroplast *trn*L^UAA^F—*trn*F^GAA^ Gene Region

Extracted genomic DNA was subjected to polymerase chain reaction (PCR) amplification (BioRad T100—Applied Biosystems^™^) using the *trn*L^UAA^F and *trn*F^GAA^ primers: 5′-CGA​AAT​CGG​TAG​ACG​CTA​CG-3′ and 5′-ATT​TGA​ACT​GGT​GAC​ACG​AG-3′ (Integrated DNA Technologies, United States), respectively, designed based on the work of Taberlet et al. (1991). The PCRs were performed in volumes of 25 µL containing 1 µL of template DNA, 12.5 µL of Qiagen Multiplex PCR Master Mix (Whitehead Scientific, South Africa), 1.5 µL of each primer (10 µM) and 8.5 µL of sterile Milli-Q H_2_0 (Ultrapure water purification system Barnstead™ MicroPure™, Thermo Fischer Scientific). The PCR reaction was performed with an initial 2-min denaturation step at 95°C. This was followed by 35 cycles, consisting of denaturation at 95°C for 1 min, annealing (adjusted from Malgas et al., 2010) at 48.6 °C for 30 s, and extension at 72°C for 40 s. The reaction was concluded with a final extension step at 72°C for 5 min (Malgas et al., 2010). Following PCR amplification, amplicons were visualised by means of agarose gel (1% w/v) electrophoresis at 110 V.

The PCR products were purified using a Sephadex A® G-50 column (Sigma Aldrich®), according to the manufacturer’s specifications, and, bidirectional sequencing reactions were performed using a BigDye™ Terminator v3.1 Cycle Sequencing Kit (Thermo Fisher Scientific), without any changes to the manufacturer’s instructions. Reactions were performed in volumes of 10 µL. Cycling conditions included an initial denaturation period of 1 min at 96°C, followed by 25 cycles of 10 s at 96°C, 5 s at 50°C, and 4 min at 60°C, as per the manufacturer’s instructions. Following this step, the sequencing reactions were sent for visualisation at the Central Analytical Facility (CAF) (DNA Sequencing Unit) at Stellenbosch University. The sequencing files were then manually trimmed on either end to a final length of 501 bp and edited using BioEdit v7.2.6.1 (Hall, 1999). The sequences of each population were then aligned in MEGA v7.0 (Kumar et al., 2016) using the ClustalW alignment algorithm with default parameters.

### Genetic Data Analyses Based on Chloroplast Gene Sequences

Diversity indices were calculated in DNASP v5.0 (Librado and Rozas, 2009) for all of the wild rooibos populations. These included the total number of haplotypes (H), haplotype diversity (*h*), nucleotide diversity (*π*), and the average number of nucleotide substitutions (*k*). A hierarchical analysis of molecular variance (AMOVA) was performed using ARLEQUIN v3.5.2 (Excoffier and Lischer, 2010) (*p* < 0.05) to investigate potential population differentiation based on the chloroplast sequences. The AMOVA tested the hypothesis of panmixia, whereby there are no restrictions between populations (global population) (F_ST_). The AMOVA also tested for genetic differentiation between the Cederberg (Western Cape) and Northern Cape regions (among regions, F_ST_), among populations within the two regions (F_SC_) as well as the genetic differentiation within populations (F_CT_). A Median-Joining haplotype network (Bandelt et al., 1999). was constructed using NETWORK v5.0.1.1 (http://www.fluxus-engineering.com), to investigate the evolutionary relationships among haplotypes.

### Nuclear SSR Amplification and Genotyping

Intact genomic DNA samples were sent to Genetic Marker Services (GMS) (United Kingdom) for the development of 18 dinucleotide microsatellite markers (Short Sequence Repeats—SSRs) specific to *Aspalathus linearis*. Sequence information was obtained from GMS for 13 developed markers (Table 2). The remaining 5 markers were not polymorphic and where therefore excluded from further analyses. The forward primers for polymorphic markers were fluorescently labelled (PET, NED, 6-FAM, TET, and VIC) by ThermoFisher (Table 2). The markers were then optimised into four multiplex groups (3–4 markers per multiplex) and amplified across a total of 86 individuals.

**TABLE 2:** Multiplex assay for 13 *Aspalathus linearis* species-specific nuclear microsatellite markers, where the repeat motif, dye, size range (bp), and annealing temperature (T_A_) are indicated.

Multiplex	Marker name	Repeat motif	Dye	Dye colour	Size range (bp)	T_A_ (°C)
1	ROI82	(AG)30	VIC	Green	180 (160–200)	58
	ROI65	(CT)17	NED	Yellow	225 (200–250)	
	ROI66	(CT)18	6-FAM	Blue	155 (130–180)	
2	ROI72B	(CT)27	TET	Green	183 (160–210)	60
	ROI70	(GA)5	PET	Red	127 (110–150)	
	ROI70B	(AG)27	NED	Yellow	200 (180–220)	
	ROI71B	(AG)36	6-FAM	Blue	250 (220–270)	
3	ROI64	(GT)14	PET	Red	150 (130–170)	58
	ROI69	(CT)5	VIC	Green	108 (90–130)	
	ROI73	(AG)12	VIC	Green	211 (190–230)	
4	ROI67	(AG)31	TET	Green	158 (140–190)	60
	ROI83	(AG)10	PET	Red	120 (100–140)	
	ROI85	(AG)8	6-FAM	Turquoise	228 (210–260)	

Polymerase chain reaction amplifications were performed in order to test for successful amplification of the markers and to optimise the PCR conditions. Each reaction consisted of a total volume of 25 µL using 1 µL of 50 ng template gDNA and 1x Qiagen Multiplex PCR Master Mix (Whitehead Scientific, South Africa). The PCR reaction was run at 95°C for 2 min as the initial denaturation step followed by 35 cycles of denaturation at 95°C for 30 s, annealing at the appropriate annealing temperature for each multiplex (Table 2) for 30 s, and an extension step at 72°C for 40 s. The reaction was completed with a final extension step at 72°C for 5 min. The amplicons were diluted 10 x with ddH_2_O and sequenced at CAF (Stellenbosch University) using the 500 LIZ® size standard. Electropherograms were analysed using the software program GeneMapper v5.0 (Applied Biosystems) for the detection of peaks, bin calling and genotyping.

### Genetic Diversity Using Nuclear Microsatellite Markers

Microsatellite genotypes were evaluated for allele stuttering, allelic dropout and the presence of null alleles while the frequency of null alleles per locus per population was calculated using MICROCHECKER v2.2.3 (Van Oosterhout et al., 2004). The software, GENEPOP ON THE WEB v4.2 (Rousset, 2008) was used to test for loci deviating from Hardy-Weinberg Equilibrium (HWE) expectations (10,000 dememorisations, 100 batches, and 10,000 iterations per batch) and for between-loci linkage disequilibrium (LD) within and across sampling populations. The inbreeding coefficient (F_IS_) for each sampling population and region (two populations from Cederberg, Western Cape, and four populations from Suid Bokkeveld, Northern Cape) were also estimated in GENEPOP. Markers under selection were then tested for in ARLEQUIN v3.5.2 (Excoffier and Lischer, 2010) (*p* < 0.05).

Genetic diversity indices were calculated for two datasets: 1) sampling populations treated separately (global dataset) and 2) sampling populations grouped into broad geographic regions (regional dataset—Cederberg vs Suid Bokkeveld). This included the average number of alleles per locus (An), the effective number of alleles per locus (Ae), allelic richness, scaled to each population size of 15 individuals (A_R_), observed and expected heterozygosity (Ho and He), Shannon’s index (I), and fixation index (F) calculated in GENALEX v6.501 (Peakall and Smouse, 2006). Polymorphism information content (PIC) of each marker was determined in MSATTOOLS v3.1.1 (Park, 2001).

### Genetic Differentiation Using Nuclear Microsatellite Markers

Principal Coordinate Analysis (PCoA) was performed in GENALEX v6.501 (Peakall and Smouse, 2006) to determine the clustering patterns across all populations. Pairwise F_ST_ estimates were calculated (999 permutations, *p* < 0.05) in order to determine the degree of genetic differentiation. A hierarchical AMOVA was performed in ARLEQUIN v3.5.2 (*p* < 0.05). The AMOVA was used to interrogate panmixia and degree of genetic differentiation as previously described (refer to *Genetic Data Analyses Based on Chloroplast Gene Sequences*). Multivariate discriminant analysis of principal components (DAPC) was performed in R Studio (R v3.5.3) using the *K*-means clustering method in the *adegenet* package to determine the genetic structure. This was achieved by the estimation of the alpha score, determining the optimal number of principal components to retain. The clustering method was run at *k* = 20. The Bayesian Information Criterion (BIC, Schwarz, 1978) was used to determine the optimal *K* value. A Bayesian clustering analysis was implemented in Structure v2.3.4 (Pritchard et al., 2000), assuming an admixture ancestry model with correlated allelic frequencies. Ten replicates were run for each *K* tested (*k* = 1–6), using a burn-in of 50,000 followed by 500,000 steps where data points were retained. The optimal *K* values were determined based on Delta *K* (Evanno et al., 2005) and the four tests of Puechmaille (2016), namely *MedMedK*, *MedMeaK*, *MaxMedK*, and *MaxMeaK*, which were determined by StructureSelector (Li and Liu 2018). Assignment plots were generated and visualised using the web service Clumpak (Kopelman et al., 2015). An assessment of relatedness (*r*) within the wild rooibos populations was performed in GENALEX v6.501, using the Queller and Goodnight (1989) estimator of relatedness (RQG). Lastly, isolation by distance (IBD) was tested using a Mantel test in GENALEX v6.501 to determine the relationship between genetic distance and geographical distance between sampling populations.

### Landscape Genetics Data Analyses Using Nuclear Microsatellite Markers

To investigate genetic stratification of wild rooibos populations as a result of landscape features, the R package *Geneland* was used (Guillot et al., 2011). First, geographic positioning system (GPS) coordinates for each of the six sampling populations from Cederberg region (Heuningvlei and Jamaka) and Northern Cape region (Blomfontein, Dobbelaarskop, Matarakopje, Melkkraal) were converted to Universal Transverse Mercator (UTM) coordinates for each sample per sampling location using the R packages *mapproj* and *PBSmapping*.

Genotypic information and UTM coordinates were used as input files for the Geneland pipeline. The population cluster range specified was *k* = 1–6, which tested the hypotheses of complete panmixia (*k* = 1) to complete isolation (*k* = 6). Allele frequencies were assumed to be correlated between populations, and a spatial model was stipulated to explain spatial patterns due to gene flow between locations. This was performed for 1,00,000 MCMC runs across 10 independent iterations.

## Results

### Genetic Diversity: Chloroplast *trn*LF Region

A total of 39 individuals from six ecologically distinct wild populations were successfully sequenced. Analysis of DNA polymorphisms revealed 5 polymorphic sites consisting of two transitions and three transversions, three of which were singletons and two were parsimony informative sites (Table 3). A total of four distinct haplotypes were observed across all populations (Tables 4, 5). The overall haplotype diversity was 0.428 and the nucleotide diversity was 0.001930. The Jamaka population (Cederberg, Western Cape) had the lowest haplotype diversity (0.286), and the lowest nucleotide diversity (0.0006) even though haplotype diversity generally varied considerably across the wild populations (*h* = 0.286–0.900). Nucleotide diversity ranged from 0.0006 to 0.010 across all the populations but the Matarakopje plants displayed the highest nucleotide diversity (Table 4). Among the populations studied, Matarakopje (Suid Bokkeveld, Northern Cape) had the highest genetic diversity.

**TABLE 3:** Polymorphic nucleotide positions of the 4 haplotypes determined across 6 wild rooibos populations.

	Nucleotide positions				
	210	218	278	355	463
Haplotypes	G	G	A	A	C
H1	.	.	.	.	.
H2	.	.	.	T	.
H3	.	.	.	.	T
H4	A	T	T	.	.

**TABLE 4:** Genetic diversity indices of collected wild rooibos populations of the chloroplast gene region, *trn*L^UAA^F—*trn*F^GAA^. *n*—sample size; H—total number of haplotypes; *h*—haplotype diversity; *π*—nucleotide diversity; *k*—average number of nucleotide substitutions.

Sampling region	*n*	H	*h*	π	*k*
Cederberg, Western Cape	**13**	**2**	**0.538**	**0.001320**	**0.538**
Heuningvlei	6	1	0.800	0.002024	1
Jamaka	7	2	0.286	0.000685	0.285
Suid Bokkeveld, Northern Cape	**26**	**3**	**0.748**	**0.004700**	**1.717**
Blomfontein	6	2	0.733	0.002554	1.266
Dobbelaarskop	8	1	0.571	0.001516	0.571
Matarakopje	5	2	0.900	0.010569	5.2
Melkkraal	7	1	0.571	0.001176	0.571
Overall	**39**	**4**	**0.428**	**0.001930**	**0.566**

Boldtype face indicates the two regions, namely the Cederberg region of the Western Cape and the Suid Bokkeveld, Northern Cape region as well as the diversity indices across all of the populations.

**TABLE 5:** Haplotype distribution of collected wild rooibos populations. *n*—number of individuals per population; H1—haplotype 1; H2—haplotype 2; H3—haplotype 3; H4—haplotype 4.

Sampling population	*n*	H1	H2	H3	H4
Blomfontein	6	3	3	0	0
Dobbelaarskop	8	8	0	0	0
Heuningvlei	6	6	0	0	0
Jamaka	7	1	0	6	0
Matarakopje	5	4	0	0	1
Melkkraal	7	7	0	0	0

**FIGURE 2:**
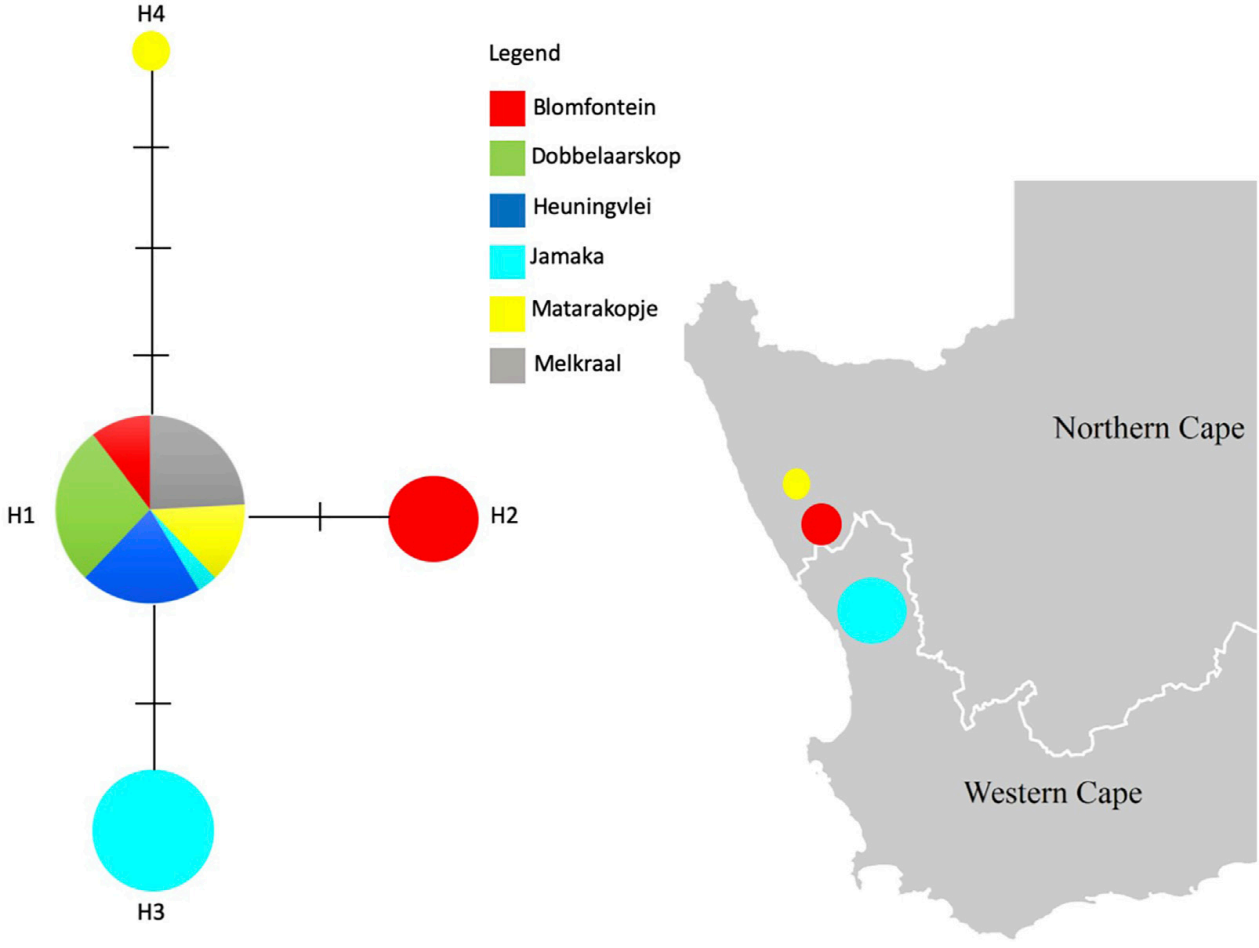
Median-Joining haplotype network based on the chloroplast intergenic *trn*L^UAA^F—*trn*F^GAA^ region for all wild rooibos populations. Dashes on the haplotype network indicate mutations. The haplotypes are shown on the map, respective to the population where they are present.

The haplotype network for the four distinct haplotypes observed in this study, appears to consist of an ancestral haplotype (H1) represented by 29 individuals across all populations whereas the remaining 3 haplotypes (H2, H3, H4) are private haplotypes representing the Blomfontein, Jamaka and Matarakopje populations, respectively (Figure 2). H2 is represented by three individuals, while H3 is represented by six individuals. It is important to note that H4 is only represented by one individual.

### Genetic Diversity: Nuclear Microsatellite Markers

In total, 86 individuals were successfully genotyped for 13 species-specific markers, with the average number of alleles ranging from 1 to 10 per marker (Supplementary Table S1). The frequencies of null alleles reached a maximum of 0.366 for locus ROI72B (Supplementary Table S1). Across all wild rooibos populations, several loci deviated from HWE, namely ROI82, ROI72B, and ROI73. The loci that were in LD include ROI65 and ROI70, ROI72B and ROI70, and lastly, ROI66 and ROI70B. Locus ROI72B showed evidence for null alleles, deviations from HWE, LD as well as being under selection (*p* < 0.05). For these reasons, this marker was excluded from further analysis. Locus ROI85 yielded little to no genotyping information, and was therefore also disregarded. Locus ROI73 presented with deviations from HWE across all populations and showed evidence of null alleles but was retained for further analysis as it did not display LD with any other markers and was also not found to be under selection. A total of 11 markers were therefore retained for further analyses. Overall, the mean number of observed alleles (An) was 6.788 while the mean number of effective alleles (Ae) was 4.217, ranging from 3.411 (Heuningvlei) to 4.966 (Matarakopje) (Table 6; Supplementary Table S1). Shannon’s information index reported an average of 1.468 and F-statistics revealed moderate genetic differentiation (F_ST_ = 0.101, *p* < 0.05). The observed heterozygosity varied from 0.528 to 0.704 while the expected heterozygosity was recorded at values of 0.618–0.723. The inbreeding coefficient (F_IS_) averaged at 0.038, indicating little to no inbreeding. Polymorphic Information Content (PIC) of the SSRs showed an average of 0.645 (Table 6). It should be noted that only 15 individuals were available per population.

**TABLE 6:** Genetic diversity indices for six wild *Aspalathus linearis* populations based on 11 microsatellite loci. *n*—sample size; PIC—Polymorphic Information Content; An—mean number of alleles per locus; Ae—mean number of effective alleles; A_R_—allelic richness; I—Shannon’s index; Ho—observed heterozygosity; He—expected heterozygosity; uHe—unbiased expected heterozygosity; F—fixation index; F_IS_—inbreeding coefficient.

	*n*	PIC	An	Ae	A_R_	I	Ho	He	uHe	F	F_IS_
Blomfontein	15	0.652	6.364	3.959	4.580	1.365	**0.528**	**0.618**	0.646	0.121	0.147
Dobbelaarskop	15	0.682	7.455	4.533	5.250	1.605	0.700	0.714	0.747	−0.018	0.019
Heuningvlei	11	0.594	5.182	**3.411**	4.250	1.287	**0.704**	0.640	0.681	−0.114	−0.100
Jamaka	15	0.606	6.909	4.217	4.640	1.418	0.565	0.636	0.661	0.077	0.113
Matarakopje	15	0.689	7.273	**4.966**	5.170	1.602	0.631	**0.723**	0.759	0.091	0.128
Melkkraal	15	0.647	7.545	4.213	5.010	1.529	0.610	0.677	0.708	0.065	0.099
Average	14.333	**0.645**	**6.788**	**4.217**	4.816	**1.468**	0.623	0.668	0.700	0.037	**0.038**

Boldtype face indicates those values discussed in text.

### Population Differentiation and Genetic Structure: Chloroplast *trn*LF Region

The hierarchical AMOVA (Table 7) highlighted genetic differentiation based on the *trn*L^UAA^F—*trn*F^GAA^ chloroplast region. Significant differentiation was observed among populations when testing for panmixia (Ф_ST_ = 0.531, *p* < 0.05), as well as when assessing differentiation among the regions (Ф_ST_ = 0.568, *p* < 0.05) and among populations within regions (Ф_SC_ = 0.480, *p* < 0.05). However, there was no significant differentiation reported within populations (Ф_CT_ = 0.168, *p* > 0.05). The AMOVA results support a divergence between regions (Cederberg and Suid Bokkeveld) as well as among populations within regions. There was no significant divergence within populations, which correlates to the presence of haplotype 1 in the majority of the total individuals that were sampled.

**TABLE 7:** Hierarchical analysis of molecular variance (AMOVA) based on *trn*L^UAA^F—*trn*F^GAA^ sequences for different structuring hypotheses of wild *Aspalathus linearis* based on six wild populations in two different sampling regions (Cederberg in the Western Cape and Suid Bokkeveld in the Northern Cape).

Hypothesis tested	Source of variation	Variation (%)	Fixation index
Panmixia	Among populations	53.15	Ф_ST_ = 0.531^a^
	Within populations	46.85	
Inter-region	Among regions	16.89	Ф_ST_ = 0.568^a^
	Among populations within regions	39.93	Ф_SC_ = 0.480^a^
	Within populations	43.18	Ф_CT_ = 0.168

a Indicates statistical significance at *p* < 0.05.

### Population Differentiation and Genetic Structure: Nuclear Microsatellite Markers

Pairwise F_ST_ estimates extended from 0.005 to 0.101 (*p* < 0.05). This indicates moderate genetic differentiation between the wild rooibos populations (Table 8; Supplementary Table S2). The largest genetic differentiation was between Heuningvlei and Jamaka (F_ST_ = 0.101), and Blomfontein and Jamaka (F_ST_ = 0.101). The hierarchical AMOVA supported this genetic differentiation across all but one level (Table 8). Significant differentiation was reported among the regions (F_ST_ = 0.064, *p* < 0.05) and among populations within regions (F_SC_ = 0.053, *p* < 0.05). However, there was no significant differentiation reported within populations (F_CT_ = 0.011, *p* > 0.05).

**TABLE 8:** Hierarchical analysis of molecular variance (AMOVA) based on 11 microsatellite markers for different structuring hypotheses of wild *Aspalathus linearis* based on six wild populations in two different sampling regions (Cederberg in the Western Cape and Suid Bokkeveld in the Northern Cape).

Hypothesis tested	Source of variation	Variation (%)	Fixation index
Panmixia	Among populations	5.897	F_ST_ = 0.058^a^
	Within populations	94.102	
Inter-region	Among regions	1.190	F_ST_ = 0.064^a^
	Among populations within regions	5.272	F_SC_ = 0.053^a^
	Within populations	93.536	F_CT_ = 0.011

a Indicates statistical significance at *p* < 0.05.

**FIGURE 3:**
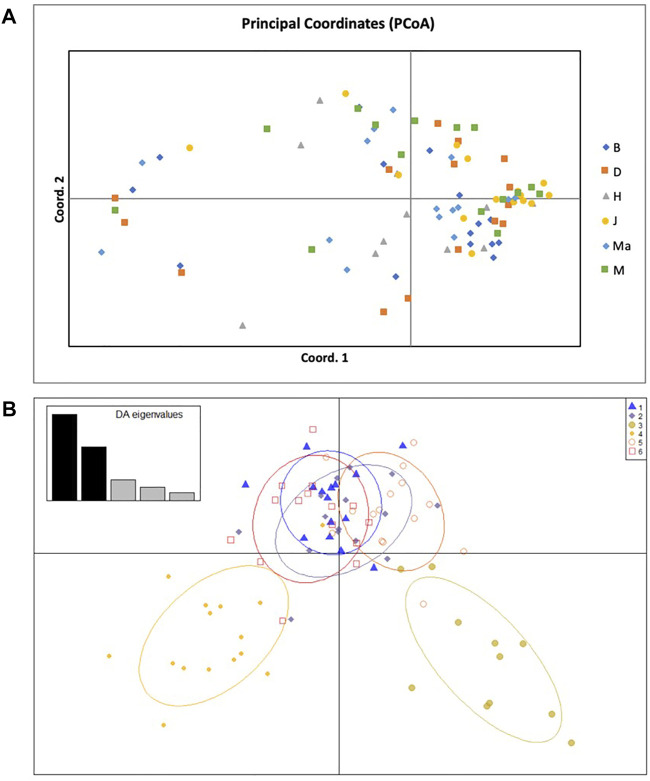
**(A)** Principal Coordinate Analysis (PCoA) of 86 individuals across six wild rooibos populations based on 11 SSR loci. B—Blomfontein, D—Dobbelaarskop, H—Heuningvlei, J—Jamaka, Ma—Matarakopje, M—Melkkraal. **(B)** Discriminant Analysis of Principal Components (DAPC) analysis of 86 individuals across six wild rooibos populations based on 11 SSR loci. B—Blomfontein, D—Dobbelaarskop, H—Heuningvlei, J—Jamaka, Ma—Matarakopje, M—Melkkraal. Each grouping represents a genetic cluster.

**FIGURE 4:**
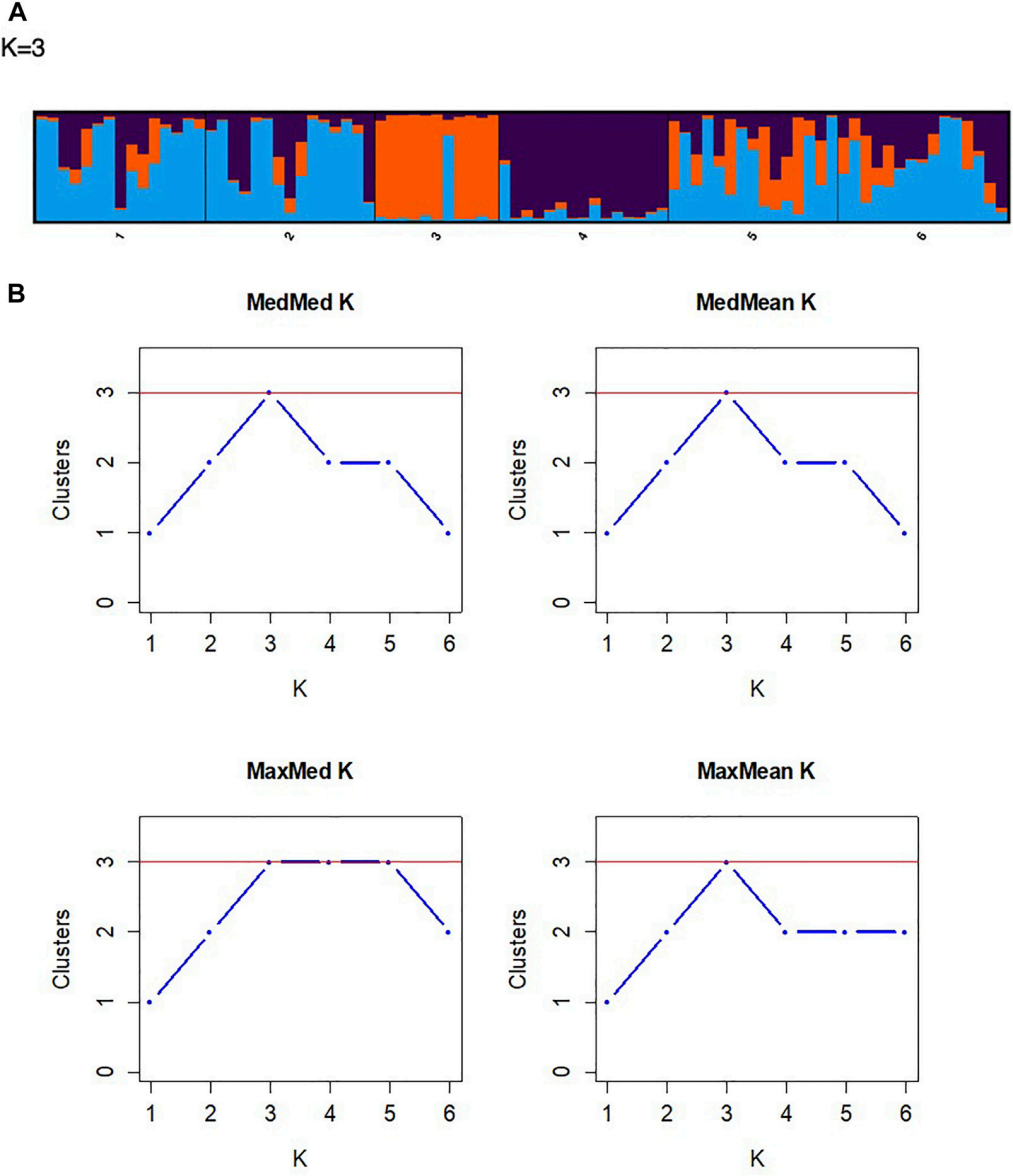
**(A)** A STRUCTURE bar plot illustrating the distribution of the collected wild rooibos populations (k = 3). **(B)** Assignment plots of the optimal K values using the four tests of Puechmaille, namely *MedMedK*, *MedMeaK*, *MaxMedK*, and *MaxMeaK.*

**FIGURE 5:**
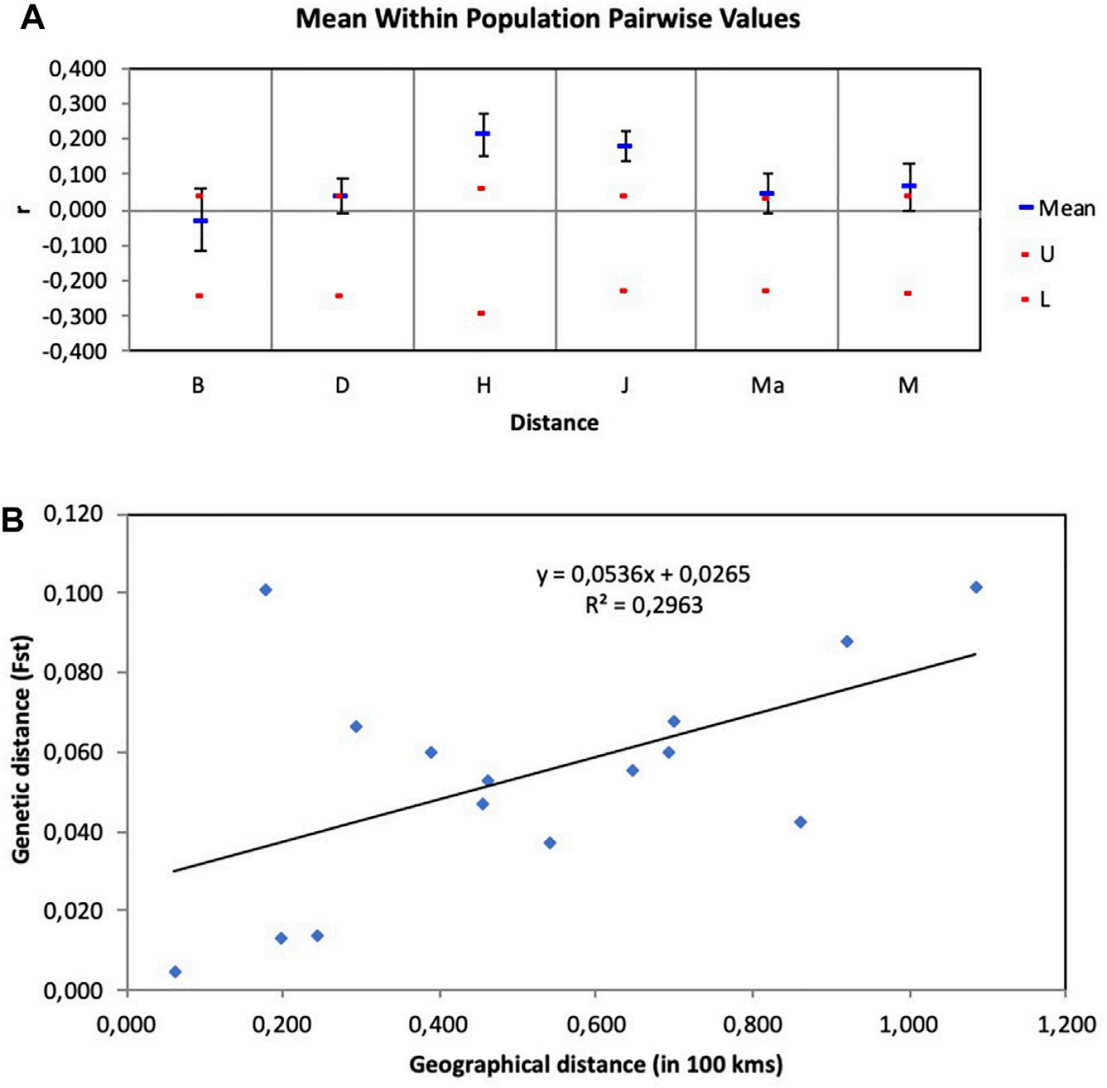
**(A)** Assessment of relatedness within wild rooibos populations. B—Blomfontein, D—Dobbelaarskop, H—Heuningvlei, J—Jamaka, Ma—Matarakopje, M—Melkkraal. U—upper confidence limit, L—lower confidence limit. **(B)** Isolation by distance (IBD) graph using genetic distance measured in F_ST_ estimates and geographical distance measured in 100 km.

The principal coordinate analysis (PCoA) revealed no patterns of genetic structure (Figure 3A), although clustering was presented by the Discriminant Analysis of Principal Components (DAPC) (Figure 3B). The two-dimensional distribution pattern observed from the PCoA (Figure 3A) totalled 13.55% variance, accumulated on the first two components (5.87 and 5.44%, respectively). The DAPC analysis revealed separation of the resprouter populations (Northern Cape populations, occurring on the positive side of PC1) versus the reseeder populations (Cederberg populations, occurring on the negative side of PC1). This was supported by the Bayesian Structure results, which showed a similar distinction between Northern Cape and Cederberg populations at *k* = 3 (Figure 4). An assessment of relatedness within the wild rooibos populations showed the highest relatedness to be within the Heuningvlei and Jamaka populations (Figure 5A).

**FIGURE 6:**
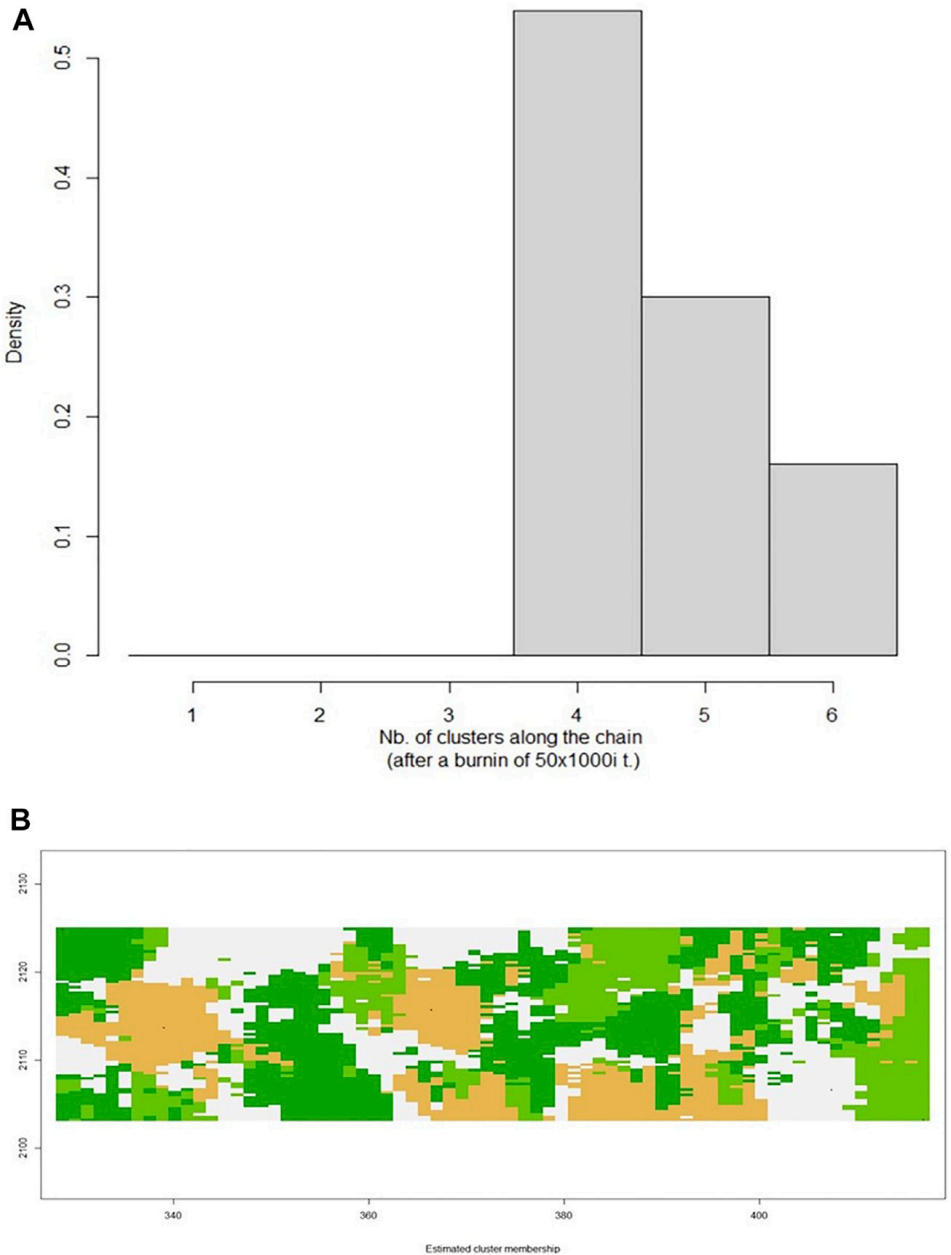
**(A)** Graphical representation of the number of clusters defined in the data set with Geneland, with k = 4 being the optimal number of clusters. **(B)** Posterior mode of population membership. Different colours denote different clusters.

The Isolation by Distance Mantel test revealed that there is a significant correlation (R^2^ = 0.296, *p* = 0.044) between genetic distance and geographical distance (Figure 5B). Additionally, based on the landscape analyses in the *Geneland* pipeline, a total of four clusters were identified (Figure 6), with Heuningvlei and Jamaka clustering independently from each other, and from the other sampling locations (Table 9). This is in congruence with the multivariate DAPC analysis, which showed a distinction between the reseeders from the Western Cape (Cederberg region) and the resprouters from the Northern Cape (Nieuwoudtville region).

**TABLE 9:** Assignment probabilities of individuals from the six geographical locations to the identified clusters.

Sampling locations	Posterior probability of population membership			
	Cluster 1	Cluster 2	Cluster 3	Cluster 4
Heuningvlei	0.111	0.148	0.074	**0.667**
Jamaka	0.185	**0.630**	0.185	-
Blomfontein	0.037	0.111	**0.741**	0.111
Dobbelaarskop	0.037	0.111	**0.741**	0.111
Matarakopje	**0.667**	0.111	-	0.222
Melkkraal	**0.667**	0.111	-	0.222

Bolded values indicate the greatest assignment probability of the four options.

**FIGURE 7:**
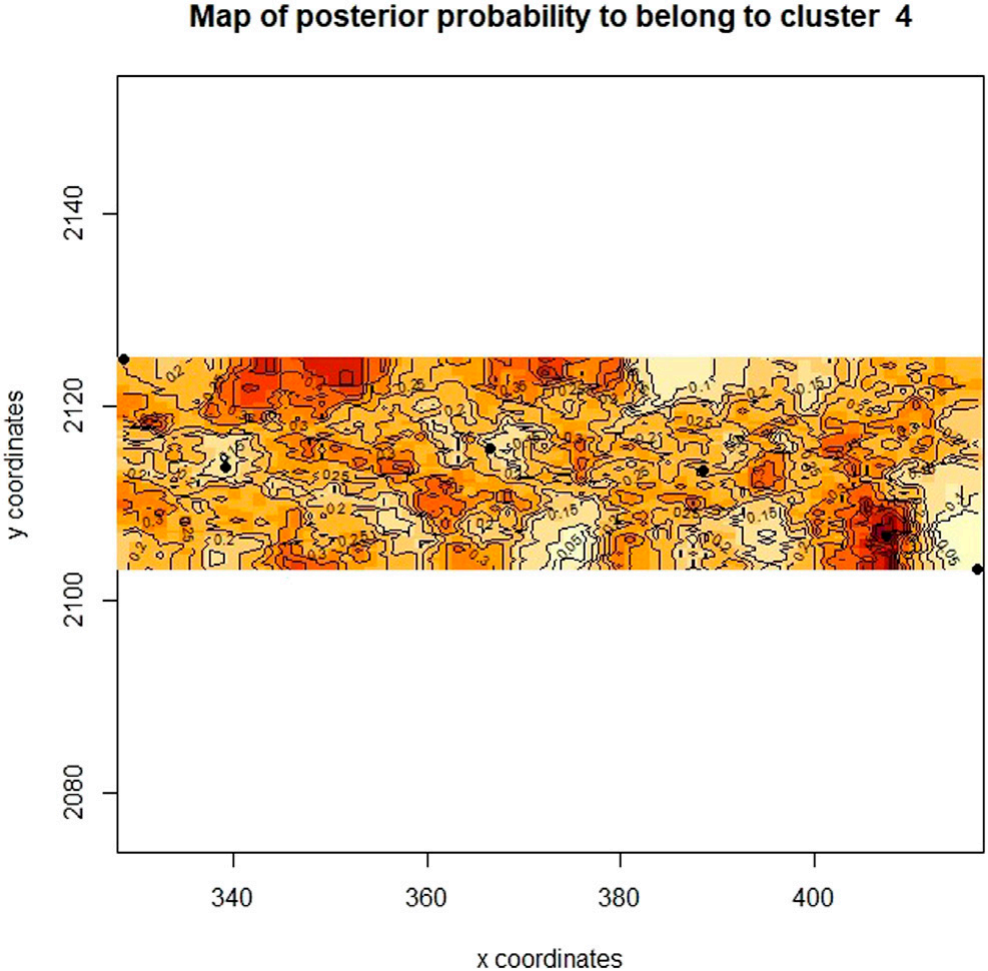
Bayesian clustering analysis displayed as a map of posterior probabilities to belong to cluster 4 (k = 4) as determined in Geneland.

Additionally, a map was constructed based on the genetic and UTM coordinate information, which unfortunately did not show clear distinctions between the previously defined clusters (Figure 7), but rather areas or zones where these clusters occur. Notably, all clusters share a degree of overlap, pointing to a level of gene flow between the groups. This was further supported by the low pairwise F_ST_ estimates between the clusters, with the Heuningvlei population in cluster four being the most distinct of the group, although none of the values were statistically significantly different between the clusters (Table 10).

**TABLE 10:** Pairwise F_ST_ estimates between clusters.

	Cluster 1	Cluster 2	Cluster 3	Cluster 4
Cluster 1				
Cluster 2	0.049			
Cluster 3	0.005	0.049		
Cluster 4	0.060	0.119	0.069	

## Discussion

### Historical Influences on Genetic Diversity and Genetic Differentiation

*Aspalathus linearis* is an extremely complex and variable species (Van der Bank et al., 1999; Van Heerden et al., 2003) and this variability is evident in the morphology, phytochemistry, ecology, and genetics with respect to wild rooibos populations (Van der Bank et al., 1999; Hawkins et al., 2011; Stander et al., 2017). The development of the fire-survival mechanisms that occur exclusively on a population level allow them to maintain their unique ecology as resprouters or reseeders. Biogeographic spatial distribution of rooibos is also associated with different chemical signatures which have been hypothesised to be associated with population-based genetic variation. In this study, rooibos was confirmed to exhibit three unique haplotypes connected to an ancestral haplotype, representing all ecotypes (Figure 2) and these data also lends support to the previous study of Malgas et al. (2010).

Overall, the haplotype and nucleotide diversity observed across both regions was low (*h* = 0.428, *π* = 0.002). Malgas et al. (2010) conducted a molecular study on wild rooibos populations using the same *trn*L^UAA^F—*trn*F^GAA^ region, but no haplotype or nucleotide diversity was presented for comparison. There is, notably, large variation in haplotype diversity and nucleotide diversity between populations and between regions (Table 4). This corresponds with the conclusions by Hawkins et al. (2011) that wild rooibos ecotypes are indeed ecologically distinct.

Rooibos may well be included in predictions of species range shifts due to climate change widely forecast for the Fynbos biome (Midgley et al., 2003; Lötter and le Maître, 2014). Recent droughts in the Western Cape of South Africa, together with soil nutrient depletion, has lowered yields from commercial rooibos production (Smith et al., 2018). Apart from this, climate change also influences wild harvesting by locals and drives patterns of wild rooibos collections especially when cultivated rooibos yields may be low and subsistence farmers become more reliant on wild populations to bulk up their yield of rooibos for trade with commercial producers (Lötter and le Maître, 2014). Across all biomes, drought and historical climate changes over time result in declining population sizes (Lötter and le Maître, 2014). Historically, during the Plio-Pleistocene (5–2.5 mya) glacial cycles, the climate became cooler, more arid and then humid and warmer between the glacial phases. This had a significant impact on the flora of Africa, particularly their ability to adapt to and survive harsh climates (Tolley et al., 2014). The chloroplast data revealed only five polymorphic sites, showing little differences between the populations, suggesting recent variability. Populations that are more genetically similar would have a more recent common ancestor as genetic variability accumulates over time (Schaal et al., 1998). It can be said that the most recent diversification event within the Cape Floristic Region, among populations within species, can be dated to the Pleistocene (Tolley et al., 2014). The diversification of wild rooibos, resulting in these four haplotypes could be hypothesised to have taken place during this time. There is however no evidence or discussion of this in previous studies.

The hierarchical AMOVA indicated significant genetic differentiation across all but one level. It revealed a high level of differentiation between regions (Cederberg and Northern Cape populations, Ф_ST_ = 0.568, *p* < 0.05) as well as among the populations within the two regions (Ф_SC_ = 0.480, *p* < 0.05). There was, however, no significant differentiation within populations (Table 7). This could be evidence of isolation between the two regions and between certain populations within the regions as many of these populations are separated by physical barriers such as the Cederberg Mountains and the chasm in the eastern part of the escarpment near Nieuwoudtville. The Cederberg Mountains span roughly 100 km, separating the Cederberg populations from the Bokkeveld plateau of the Northern Cape. The Cederberg region forms part of the Cape Fold Mountain range and thus acts as a physical barrier with a channelling effect, restricting and/or limiting dispersal routes. There are also several rivers, namely the Olifants River in the Cederberg area and the Doring River spanning from the Cederberg to the Suid Bokkeveld area (Malgas et al., 2010). These rivers are also important to consider as genetic barriers (Davis et al., 2018). More significantly, the chasm that separates the Dobbelaarskop population from the other Northern Cape populations in the Suid Bokkeveld should be considered as another genetic barrier.

Within this Cape Floristic Region, ants are often responsible for seed dispersal, as with rooibos. This has a significant effect on gene flow. Ants have a relatively low dispersal capacity, only having a range of a few meters (Lötter and le Maître, 2014). This possibly contributes to limited gene flow, which in turn creates genetic structuring between populations that are separated by large physical distances. Habitat specificity also drives local colonisation and this may lead to geographically distinct clades (Wang et al., 2019). *Aspalathus linearis* is known to be a strict endemic and major historical losses of habitat as a result of anthropogenic activities may have led to population contractions, leading to highly fragmented wild populations of rooibos that are low in plant numbers, also contributing to shaping genetic variation and spatial genetic patterns (Hawkins et al., 2011).

The threat to genetic diversity is exacerbated by environmental change and the conversion of wild rooibos habitats for agricultural use. It can be argued that the local mainstream rooibos industry does not necessarily place much value on the wild rooibos (Lötter and le Maitre, 2014; Wynberg, 2017) and as a result, commercial land-users are under economic pressure to cultivate rooibos as their main source of income. For small-scale resource-poor farmers, economic pressures are worse, but over-exploitation is circumvented in two ways. First, wild rooibos is highly valued in niche overseas markets, fetching premium prices that help to offset the profit that would have been made from land conversion and that would have come at the cost of wild rooibos habitats (Malgas et al., 2010). Secondly, the Heiveld Co-operative holds its members strictly accountable for conservation and husbandry of wild rooibos, cultivating an ethics of care for populations in the wild that include co-occurring species and biodiversity in general. In these organisations, the genetic diversity of wild rooibos is also valued. Farmers know from their own local ecological knowledge that wild rooibos is more resilient to climate change, pests and disease (Louw, 2006). Malgas et al. (2010) suggested that some of these distinct population morphotypes and/or chemotypes that were prominent in some areas in the past may no longer exist in the present. This loss of diversity impacts genetic variability and has the potential to lead to the fixation of alleles, reduced adaptability to environmental stressors and heightens the chances of inbreeding.

Malgas et al. (2010) discussed the correspondence between molecular analyses and morphology-based analyses on geographical locations when investigating haplotypic variation. Because *A. linearis* is highly specialised in its spatial distribution, which contributes to its phenology, molecular physiology, the microenvironment and other ecosystem-driven attributes, it may thus have played a greater contribution to adaptive evolutionary genetic traits. Often, phylogeographical patterns are dynamic in nature and are continually being influenced by adaptive potential, ecological interactions, and climate change (Tolley et al., 2014).

### Contemporary Influences on Rooibos Genetic Diversity and Genetic Differentiation

To the best of our knowledge, no previous assessment of genetic diversity or population structure using microsatellite markers has been conducted in *Aspalathus linearis*. Previous genetic studies on this species utilised isozymes (Van der Bank et al., 1995; Van der Bank et al., 1999), as well as chloroplast sequencing and a single nuclear region (Malgas et al., 2010), focusing on the evolution of resprouters versus reseeders, haplotypic variation, and phylogenetic relationships. This current study found a higher ratio of observed number of alleles (An) to effective number of alleles (Aee). This could indicate that the alleles represented across all of the sampled populations are quite variable between populations and not all alleles are present in every population. A low-to-moderate genetic diversity was found based on the diversity indices of the wild rooibos populations (Table 7; Wang et al., 2019). Lower genetic diversity could be explained by species with small population sizes and this concurs with the observation that some of the collected wild rooibos populations were, noticeably, growing as small and patchy populations. This could be due to seasonal changes, which could lead to overexploitation when populations are thriving. Such practices are likely to continue unabated in the future and this particular study also serves to highlight the urgent need for the conservation of unique genetic populations of rooibos. Biological characteristics, reproductive ecology, and geography are key factors that influence genetic diversity (Ellegren and Galtier, 2016; Selseleh et al., 2019) and those plants that are found to occur as small populations with a sporadic distribution may thus show limited genetic variation as individual plants in isolation are thus likely to reproduce with each other. It is important to note that low genetic diversity could also be owing to the number of individuals that were collected. Additionally, low genetic diversity may be associated with high levels of relatedness between certain populations (Table 8; Figure 6A). For this reason, inbreeding (F_IS_) was investigated but little to no inbreeding was detected in the populations focused on in this study (Table 5; F_IS_ = 0.038).

Gene flow is directly influenced through seed dispersal and pollinators (Schaal et al., 1998) and spatial connectedness of populations may thus be important for pollination where insects are the main pollinators. Pollination, in particular, plays a fundamental role in species diversity and cross-pollinated plants have more genetic variation than those plants that are self-pollinated. Rooibos is dependent on flying pollinators and ants for seed dispersal (Herbst, 2011; Lötter and le Maître, 2014; Melin et al., 2014). Although flying pollinators have the potential to reach further distances, seeds that are dispersed by ants may be limited in their dispersal mechanisms (Lötter and le Maître, 2014). Intrinsic genetic variation enables plants to respond to changing environmental conditions and large seasonal variation and as a result, fluctuation in pollination patterns. Micro-climates are known to contribute to localised adaptations that display epigenetic changes and these traits become heritable over time (Malgas et al., 2010; Kronholm and Collins, 2016).

The Heuningvlei and Jamaka populations showed the highest relatedness within populations (Figure 6A) and were also the most differentiated according to the F_ST_ estimates (Table 6). These two populations both occur within the Cederberg Mountain range, are located only 18 km apart, yet spatially separated by these mountains, with the Heuningvlei population situated in a valley. It may thus be expected that higher levels of gene flow are likely to occur. As these populations occur in closer proximity, greater genetic exchange between these two populations would thus be expected and the Cederberg Mountains represent a geographic boundary, isolating these populations from the Northern Cape group into a distinct lineage. The landscape genetic analysis performed in this study (Geneland cluster analysis and IBD Mantel test) supports the restriction of gene exchange resultant from these vast regions with geographic boundaries. The overlapping areas in posterior probabilities of population membership allude to the possibility of barriers to gene flow, and these coincide with the Cederberg Mountainous region between the two sampling regions, Cederberg and Nieuwoudtville (Figure 7). Furthermore, the microclimates between Heuningvlei and Jamaka are more similar in comparison to those plants found in the Northern Cape where the average temperature is lower and more rainfall occurs than in the Cederberg. Other factors that may be considered as being important to drive greater genetic relatedness within the Heuningvlei and Jamaka groups may be the influence of reproductive ecology, particularly reproductive barriers and the status of pollination and seed dispersal. In fact, reproductive barriers are known to limit gene flow, greatly influencing population structure and support genetic differentiation (Schaal et al., 1998). These two Cederberg populations are genetically differentiated (F_SC_ = 0.053, *p* < 0.05), most likely as a result of physical isolation, despite some obvious similarities in terms of their morphological appearance and chemical profiles (Stander et al., 2017). This results in distinct populations which are often overexploited, reducing the number of individuals within the population itself. The limited gene flow can influence genetic variation and over time, a high level of relatedness within these populations could result, as seen in this present study.

Available scientific information regarding the reproductive ecology associated with rooibos is tenuous. As far as we are aware, the exact plant-pollinator networks for *Aspalathus* remain ill-defined but wasps and bees are thought to be the important animals for pollinating rooibos although no study has focused on this directly (Gess, 2000; Herbst, 2011). For this reason, it thus becomes more difficult to explain likely effects linked to genetic structure based on a reproductive ecology context. Wild rooibos populations do not display both mechanisms of fire survival strategy; these mechanisms are mutually exclusive on a population level (Van der Bank et al., 1999). Because rooibos populations display either reseeding or resprouting mechanisms for the vegetative establishment of new plants, various adaptations such as this, may also then influence intra- and inter-population dynamics at local and regional scales.

Our data suggests that genetic diversity of wild rooibos populations decline along a gradient, from Melkkraal in the north to Jamaka in the south (Figure 1). This may contribute to the understanding that the Cederberg is the center of endemism of rooibos and that the populations in the Suid Bokkeveld have radiated out of the Cederberg over time explaining the increased diversity in the Northern Cape. This data illustrates the potential of clinal variation through gradual variation of a trait being inherited over time across a geographic gradient though there is not sufficient evidence to substantiate this claim. This geographical gradient could be altitude, climate or other environmental influences. Additionally, it is important to consider that these two regions have different biomes and the transition zones between them could result in increased species richness and thus could explain the increased diversity in the Northern Cape populations. Clinal variation could imply restricted gene flow and results in phenotypic diversity (Takahashi et al., 2019) and in the case of rooibos, interpopulation metabolomic differences (Stander et al., 2017). Similar results were observed by Van der Bank et al. (1999), where it was inferred that speciation would be more likely to occur in reseeder populations and that resprouters have a higher possibility for clinal variation. In this current study, all the populations investigated in the Suid Bokkeveld are resprouters and the Cederberg populations are reseeders even though resprouters do occur in the Cederberg.

There was a significant correlation between geographical distance and genetic distance (Figure 5B). It is important to consider spatial differences as well as geographical barriers and how that might influence seed dispersal and pollination and its subsequent contribution to genetic population structure. Seasonal variation also largely determines the distribution of flying pollinators. Rooibos is typically pollinated from September to November with a few still flowering in January (Malgas et al., 2011). It is possible that these wild populations flower at different times and flowering may not always be synchronised amongst diverse population groups, and that is related to the microclimates or specific locality where these unique populations are found (Joubert et al., 2008). Differences in flowering strategies are likely to influence the likelihood of reproduction between these ecotypes. The intensification of agriculture has caused significant changes to the landscape of both the Western Cape and Northern Cape over time, leading to major biodiversity losses for both plants and animals (Vlok and Raimondo, 2011; Schutte-Vlok and Raimondo, 2020). The wild plants and the ecosystems of the Cape floral region are vastly different from what was observed in the past, and natural plant stands that act as refugia for insect pollinators are patchy and fragmented with continuous habitats that once were in existence, no longer available (Tolley et al., 2014). This has an impact on foraging distances for pollinators and unfortunately alters dispersal mechanisms as traveling distances for nesting, and nutrient resources become further apart. The connection of ecosystems to each other becomes less possible and so geospatial distance can in such a way influence the genetic makeup of plants of the same species.

Pairwise F_ST_ analyses revealed moderate genetic differentiation whereas DAPC analysis determined genetic structure across all sampled populations. These results show a high level of congruence between the genetic data sets, confirming the genetic patterns resolved using the chloroplast intergenic region (Table 5). The DAPC is a powerful multivariate approach to resolve the number of genetic clusters that are present between the populations without prior knowledge of their genetic relationship and thus reduces population bias. The DAPC plot reveals genetic structure without the assumption that the populations are panmictic (Jombart et al., 2010). In this study, the DAPC indicated that the reseeder populations (Jamaka and Heuningvlei ecotypes) are genetically distinct from the Northern Cape resprouter populations (Figure 3B). These fire survival strategies may limit genetic hybridisation; leading to population-isolated types that express particular phenotypes. The DAPC also supports the moderate genetic differentiation that is evident from the pairwise F_ST_ analysis. Additionally, the Bayesian structure analysis revealed the separation of populations into three clusters, the Northern Cape populations clustering together, and the two Western Cape populations clustered individually as distinct populations (k = 3; Figure 4). This consolidates the DAPC, strengthening the evidence of reseeders and resprouters being genetically distinct. This finding is not necessarily new as similar results have been reported by Van der Bank et al. (1999) but it serves to corroborate that particular study which was based on isozymes. Van der Bank et al. (1999) proposed that resprouters are derived from reseeders. Unfortunately, that study did not mention the evolutionary time period whereby this diversification might have occurred. It is interesting that this proposed separation of reseeders and resprouters has been maintained in present times. Historical events such as population bottlenecks are strong indicators of genetic structure (Schaal et al., 1998; Davis et al., 2018). The separation of resprouters versus reseeders highlights the potential effects of genetic drift, isolation by distance, fire survival strategies, and environmental differences between the region sampled as well as the combination of all of these factors and how these could be responsible for the populations decreasing in numbers and may need to be investigated further (Clarke et al., 2013). Furthermore, the DAPC showed separation of the two Cederberg populations (Western Cape), corroborating pairwise F_ST_ values (Table 6), and likely indicating that they are genetically distinct. This is interesting because although these populations are in close proximity to each other, the biogeographic landscape may create a channelling effect, limiting extensive genetic exchange. This could be due to the geographical barrier between these two populations as the Cederberg Mountains create a terrain of valleys and peaks with the Heuningvlei population found in a deep valley. Moreover, these two populations are also morphologically different. The Jamaka population was particularly unique as it was smaller in size compared to most plants and had blue-green coloured leaves as opposed to bright green. The presence of three main clusters is clearly reflected in the DAPC, based on the current suite of microsatellite markers. The inclusion of additional microsatellites could potentially lead to higher resolution in terms of population structure at the intra-regional level.

## Conclusion

Haplotype divergence of the ecotypes from the Cederberg and Suid Bokkeveld provided insights into the genetic history of these populations and there was a clear separation between resprouters and reseeders corroborating the original hypothesis. Through using both nuclear markers and chloroplast sequencing, a comprehensive and complementary portrait of the genetic structure of wild rooibos was evident and there was an ancestral haplotype consisting of both reseeders and resprouters. This data may be indicative of clinal genetic variation that suggests decreased diversity from the Suid Bokkeveld populations down into the Cederberg region. Overall, low intra-specific population diversity was strongly evident in wild collected rooibos, particularly in the reseeder populations of Jamaka and Heuningvlei. Wild rooibos populations occur as sparse collections with few individuals and limited options for allowing frequent gene flow, making them more susceptible to biodiversity loss. The results presented herein thus highlight the importance of assessing genetic variability and the need for implementing strategies for conservation priorities for rooibos that is a highly restricted endemic growing in biogeographic regions that face both habitat degradation and future climate changes. In this particular context, the correct and appropriate management of wild genetic *A. linearis* resources is thus strongly encouraged as distinct gene pools have been confirmed in this study. Many land-users have commented on the population decline over the last few decades and these knowledge-holders have emphasised that more recently it is becoming more difficult every year to find a suitable harvest of wild populations due to populations declining. Conservation initiatives may prove to be of value for both *in situ* and *ex situ* strategies. Additionally, it is important to prioritise conservation efforts at every step of the supply chain, particularly for such a uniquely endemic species such as rooibos. Conservation strategies could include thorough monitoring and record-keeping of wild harvesting that supports livelihoods as well as deposits of wild populations to a gene bank for the conservation of distinct populations.

## Data Availability

The data presented in this study are deposited in NCBI, accession numbers OK771546-OK771592 (trnLF chloroplast sequences) and OL438920-OL438931 (nuclear microsatellite sequences).
