# Supplementary material for: Resprouters Versus Reseeders: Are Wild Rooibos Ecotypes Genetically Distinct?
Source: Front Genet. 2021 Dec 20;12:761988. doi: 10.3389/fgene.2021.761988 (PMC8721207; doi:10.3389/fgene.2021.761988)
Supplement: Supplementary file 1 [file Table1.docx]

# SUPPLEMENTARY MATERIAL

**Table S1** Genetic diversity indices per microsatellite marker for each wild rooibos population. These estimates include polymorphic information content - PIC; average number of alleles - Na; effective number of alleles - Ne; Shannon’s information index - I; observed heterozygosity - HO; fixation index - F. The mean and standard error (SE) for each diversity estimate is provided below each population. An asterisk (*) indicates deviation from Hardy-Weinberg equilibrium (*P* < 0.05).

| **Pop** | **Locus** | **PIC** | **Na** | **Ne** | **I** | **Ho** | **He** | **uHe** | **F** |
| --- | --- | --- | --- | --- | --- | --- | --- | --- | --- |
| **Blomfontein** | Loc82* | 0,821 | 12,000 | 8,963 | 2,347 | 0,455 | 0,888 | 0,931 | 0,488 |
|  | Loc65 | 0,860 | 4,000 | 1,502 | 0,692 | 0,231 | 0,334 | 0,348 | 0,310 |
|  | Loc66* | 0,804 | 5,000 | 2,824 | 1,231 | 0,500 | 0,646 | 0,674 | 0,226 |
|  | Loc70 | 0,212 | 3,000 | 1,291 | 0,456 | 0,250 | 0,226 | 0,236 | -0,108 |
|  | Loc70B* | 0,535 | 3,000 | 2,571 | 1,011 | 0,500 | 0,611 | 0,638 | 0,182 |
|  | Loc71B | 0,821 | 11,000 | 6,145 | 2,095 | 0,769 | 0,837 | 0,871 | 0,081 |
|  | Loc64 | 0,823 | 9,000 | 6,261 | 2,011 | 0,917 | 0,840 | 0,877 | -0,091 |
|  | Loc69 | 0,164 | 2,000 | 1,220 | 0,325 | 0,200 | 0,180 | 0,186 | -0,111 |
|  | Loc73* | 0,661 | 6,000 | 3,375 | 1,442 | 0,333 | 0,704 | 0,745 | 0,526 |
|  | Loc67* | 0,652 | 6,000 | 3,349 | 1,415 | 0,833 | 0,701 | 0,732 | -0,188 |
|  | Loc83 | 0,816 | 9,000 | 6,050 | 1,985 | 0,818 | 0,835 | 0,874 | 0,020 |
|  | **Mean** | **0,652** | **6,364** | **3,959** | **1,365** | **0,528** | **0,618** | **0,646** | **0,121** |
|  | **SE** | **0,072** | **0,981** | **0,728** | **0,199** | **0,076** | **0,074** | **0,077** | **0,071** |
| **Dobbelaarskop** | Loc82 | 0,806 | 11,000 | 5,633 | 2,051 | 0,692 | 0,822 | 0,855 | 0,158 |
|  | Loc65 | 0,812 | 13,000 | 5,647 | 2,181 | 0,500 | 0,823 | 0,859 | 0,392 |
|  | Loc66 | 0,739 | 8,000 | 4,235 | 1,735 | 0,667 | 0,764 | 0,797 | 0,127 |
|  | Loc70 | 0,432 | 4,000 | 1,988 | 0,882 | 0,692 | 0,497 | 0,517 | -0,393 |
|  | Loc70B | 0,501 | 4,000 | 2,410 | 1,013 | 0,800 | 0,585 | 0,616 | -0,368 |
|  | Loc71B | 0,802 | 9,000 | 5,586 | 1,956 | 0,778 | 0,821 | 0,869 | 0,053 |
|  | Loc64 | 0,821 | 9,000 | 5,930 | 1,955 | 0,923 | 0,831 | 0,865 | -0,110 |
|  | Loc69 | 0,239 | 2,000 | 1,385 | 0,451 | 0,333 | 0,278 | 0,287 | -0,200 |
|  | Loc73* | 0,769 | 6,000 | 5,128 | 1,713 | 0,400 | 0,805 | 0,847 | 0,503 |
|  | Loc67 | 0,718 | 6,000 | 4,114 | 1,549 | 0,917 | 0,757 | 0,790 | -0,211 |
|  | Loc83 | 0,859 | 10,000 | 7,806 | 2,174 | 1,000 | 0,872 | 0,913 | -0,147 |
|  | **Mean** | **0,682** | **7,455** | **4,533** | **1,605** | **0,700** | **0,714** | **0,747** | **-0,018** |
|  | **SE** | **0,058** | **0,965** | **0,559** | **0,166** | **0,062** | **0,053** | **0,056** | **0,084** |
| **Heuningvlei** | Loc82 | 0,813 | 9,000 | 5,902 | 1,983 | 0,909 | 0,831 | 0,870 | -0,095 |
|  | Loc65 | 0,641 | 6,000 | 3,115 | 1,407 | 0,556 | 0,679 | 0,719 | 0,182 |
|  | Loc66 | 0,627 | 5,000 | 3,125 | 1,305 | 0,800 | 0,680 | 0,716 | -0,176 |
|  | Loc70 | 0,365 | 2,000 | 1,923 | 0,673 | 0,800 | 0,480 | 0,505 | -0,667 |
|  | Loc70B | 0,563 | 3,000 | 2,778 | 1,055 | 0,400 | 0,640 | 0,711 | 0,375 |
|  | Loc71B | 0,540 | 5,000 | 2,500 | 1,139 | 0,800 | 0,600 | 0,632 | -0,333 |
|  | Loc64 | 0,631 | 6,000 | 3,048 | 1,386 | 1,000 | 0,672 | 0,717 | -0,488 |
|  | Loc69 | 0,152 | 2,000 | 1,198 | 0,305 | 0,182 | 0,165 | 0,173 | -0,100 |
|  | Loc73* | 0,631 | 6,000 | 3,048 | 1,386 | 0,500 | 0,672 | 0,717 | 0,256 |
|  | Loc67 | 0,758 | 6,000 | 4,762 | 1,650 | 0,800 | 0,790 | 0,832 | -0,013 |
|  | Loc83 | 0,816 | 7,000 | 6,125 | 1,871 | 1,000 | 0,837 | 0,901 | -0,195 |
|  | **Mean** | **0,594** | **5,182** | **3,411** | **1,287** | **0,704** | **0,640** | **0,681** | **-0,114** |
|  | **SE** | **0,054** | **0,588** | **0,428** | **0,135** | **0,072** | **0,052** | **0,055** | **0,086** |
| **Jamaka** | Loc82* | 0,897 | 14,000 | 10,465 | 2,480 | 0,733 | 0,904 | 0,936 | 0,189 |
|  | Loc65 | 0,886 | 13,000 | 9,561 | 2,395 | 0,929 | 0,895 | 0,929 | -0,037 |
|  | Loc66* | 0,705 | 7,000 | 3,881 | 1,567 | 0,571 | 0,742 | 0,770 | 0,230 |
|  | Loc70 | 0,376 | 5,000 | 1,654 | 0,841 | 0,467 | 0,396 | 0,409 | -0,180 |
|  | Loc70B | 0,500 | 4,000 | 2,215 | 1,013 | 0,417 | 0,549 | 0,572 | 0,241 |
|  | Loc71B* | 0,837 | 10,000 | 6,818 | 2,080 | 0,600 | 0,853 | 0,883 | 0,297 |
|  | Loc64 | 0,474 | 6,000 | 1,988 | 1,077 | 0,538 | 0,497 | 0,517 | -0,083 |
|  | Loc69 | 0,204 | 2,000 | 1,301 | 0,393 | 0,267 | 0,231 | 0,239 | -0,154 |
|  | Loc73* | 0,634 | 5,000 | 3,077 | 1,333 | 0,200 | 0,675 | 0,711 | 0,704 |
|  | Loc67 | 0,540 | 4,000 | 2,465 | 1,077 | 0,643 | 0,594 | 0,616 | -0,082 |
|  | Loc83 | 0,617 | 6,000 | 2,965 | 1,345 | 0,846 | 0,663 | 0,689 | -0,277 |
|  | **Mean** | **0,606** | **6,909** | **4,217** | **1,418** | **0,565** | **0,636** | **0,661** | **0,077** |
|  | **SE** | **0,063** | **1,102** | **0,929** | **0,189** | **0,064** | **0,061** | **0,063** | **0,082** |
| **Matarakopje** | Loc82* | 0,927 | 17,000 | 14,519 | 2,750 | 0,643 | 0,931 | 0,966 | 0,310 |
|  | Loc65* | 0,794 | 8,000 | 5,452 | 1,862 | 0,462 | 0,817 | 0,849 | 0,435 |
|  | Loc66 | 0,640 | 5,000 | 3,273 | 1,316 | 0,417 | 0,694 | 0,725 | 0,400 |
|  | Loc70 | 0,587 | 5,000 | 2,649 | 1,253 | 0,714 | 0,622 | 0,646 | -0,148 |
|  | Loc70B | 0,633 | 6,000 | 3,184 | 1,373 | 0,909 | 0,686 | 0,719 | -0,325 |
|  | Loc71B* | 0,833 | 9,000 | 6,627 | 2,034 | 0,462 | 0,849 | 0,883 | 0,456 |
|  | Loc64 | 0,811 | 9,000 | 5,930 | 1,961 | 0,923 | 0,831 | 0,865 | -0,110 |
|  | Loc69 | 0,262 | 2,000 | 1,451 | 0,490 | 0,385 | 0,311 | 0,323 | -0,238 |
|  | Loc73* | 0,730 | 6,000 | 4,263 | 1,586 | 0,222 | 0,765 | 0,810 | 0,710 |
|  | Loc67 | 0,663 | 8,000 | 3,429 | 1,530 | 1,000 | 0,708 | 0,739 | -0,412 |
|  | Loc83 | 0,701 | 5,000 | 3,846 | 1,471 | 0,800 | 0,740 | 0,822 | -0,081 |
|  | **Mean** | **0,689** | **7,273** | **4,966** | **1,602** | **0,631** | **0,723** | **0,759** | **0,091** |
|  | **SE** | **0,050** | **1,107** | **1,008** | **0,163** | **0,074** | **0,047** | **0,049** | **0,109** |
| **Melkkraal** | Loc82* | 0,789 | 12,000 | 5,121 | 2,053 | 0,538 | 0,805 | 0,837 | 0,331 |
|  | Loc65* | 0,902 | 14,000 | 10,903 | 2,522 | 0,615 | 0,908 | 0,945 | 0,322 |
|  | Loc66 | 0,509 | 6,000 | 2,167 | 1,139 | 0,538 | 0,538 | 0,560 | 0,000 |
|  | Loc70 | 0,439 | 8,000 | 1,822 | 1,088 | 0,533 | 0,451 | 0,467 | -0,182 |
|  | Loc70B | 0,513 | 5,000 | 2,414 | 1,076 | 0,538 | 0,586 | 0,609 | 0,081 |
|  | Loc71B* | 0,801 | 8,000 | 5,633 | 1,885 | 0,538 | 0,822 | 0,855 | 0,345 |
|  | Loc64 | 0,727 | 8,000 | 3,967 | 1,729 | 0,909 | 0,748 | 0,784 | -0,215 |
|  | Loc69 | 0,262 | 2,000 | 1,451 | 0,490 | 0,385 | 0,311 | 0,323 | -0,238 |
|  | Loc73* | 0,657 | 5,000 | 3,379 | 1,376 | 0,286 | 0,704 | 0,758 | 0,594 |
|  | Loc67 | 0,756 | 9,000 | 4,612 | 1,789 | 0,929 | 0,783 | 0,812 | -0,186 |
|  | Loc83 | 0,765 | 6,000 | 4,878 | 1,670 | 0,900 | 0,795 | 0,837 | -0,132 |
|  | **Mean** | **0,647** | **7,545** | **4,213** | **1,529** | **0,610** | **0,677** | **0,708** | **0,065** |
|  | **SE** | **0,055** | **0,965** | **0,759** | **0,162** | **0,061** | **0,053** | **0,055** | **0,083** |

**Table S2** Pairwise F_ST_ estimates between wild rooibos populations. F_ST_ values shown below the diagonal. Probability, based on 9999 permutations is shown above the diagonal. B – Blomfontein; D – Dobbelaarskop; H – Heuningvlei; J – Jamaka; Ma – Matarakopje; M – Melkkraal.

| **B** | **D** | **H** | **J** | **Ma** | **M** |  |
| --- | --- | --- | --- | --- | --- | --- |
|  | 0,001 | 0,000 | 0,000 | 0,000 | 0,000 | **B** |
| 0,047 |  | 0,000 | 0,000 | 0,241 | 0,062 | **D** |
| 0,088 | 0,052 |  | 0,000 | 0,002 | 0,000 | **H** |
| 0,101 | 0,056 | 0,101 |  | 0,000 | 0,000 | **J** |
| 0,060 | 0,005 | 0,037 | 0,068 |  | 0,054 | **Ma** |
| 0,067 | 0,014 | 0,060 | 0,042 | 0,013 |  | **M** |
